# Towards a Near Infrared Spectroscopy-Based Estimation of Operator Attentional State

**DOI:** 10.1371/journal.pone.0092045

**Published:** 2014-03-14

**Authors:** Gérard Derosière, Sami Dalhoumi, Stéphane Perrey, Gérard Dray, Tomas Ward

**Affiliations:** 1 Movement to Health (M2H), EuroMov, Montpellier-1 University, Montpellier, France; 2 Biomedical Engineering Research Group (BERG), National University of Ireland Maynooth (NUIM), Maynooth, Ireland; 3 Laboratoire de Génie Informatique et d’Ingénierie de Production (LG2IP), Ecole des Mines d’Alès, Nîmes, France; Tokyo Metropolitan Institute of Medical Science, Japan

## Abstract

Given the critical risks to public health and safety that can involve lapses in attention (e.g., through implication in workplace accidents), researchers have sought to develop cognitive-state tracking technologies, capable of alerting individuals engaged in cognitively demanding tasks of potentially dangerous decrements in their levels of attention. The purpose of the present study was to address this issue through an investigation of the reliability of optical measures of cortical correlates of attention in conjunction with machine learning techniques to distinguish between states of full attention and states characterized by reduced attention capacity during a sustained attention task. Seven subjects engaged in a 30 minutes duration sustained attention reaction time task with near infrared spectroscopy (NIRS) monitoring over the prefrontal and the right parietal areas. NIRS signals from the first 10 minutes of the task were considered as characterizing the *‘full attention*’ class, while the NIRS signals from the last 10 minutes of the task were considered as characterizing the ‘*attention decrement*’ class. A two-class support vector machine algorithm was exploited to distinguish between the two levels of attention using appropriate NIRS-derived signal features. Attention decrement occurred during the task as revealed by the significant increase in reaction time in the last 10 compared to the first 10 minutes of the task (p<.05). The results demonstrate relatively good classification accuracy, ranging from 65 to 90%. The highest classification accuracy results were obtained when exploiting the oxyhemoglobin signals (i.e., from 77 to 89%, depending on the cortical area considered) rather than the deoxyhemoglobin signals (i.e., from 65 to 66%). Moreover, the classification accuracy increased to 90% when using signals from the right parietal area rather than from the prefrontal cortex. The results support the feasibility of developing cognitive tracking technologies using NIRS and machine learning techniques.

## Introduction

Attention to a cognitively demanding task cannot be maintained at a high level indefinitely. During a sustained attention task, as time elapses, the level of attention progressively diminishes negatively impacting task performance [1]. Lapses in attention are behaviorally characterized by an increase in reaction time (RT; e.g., [2]) a phenomenon that can impact severely on activities of daily living. For instance, work-related injuries [3], [4] and traffic accidents [5] are typical consequences of attention decrement. Recently, researchers have sought to develop cognitive tracking technologies capable of alerting users to such degradation in their attention levels [6], [7]. The aspiration is that such technology can facilitate optimal human-machine interactions in real-life settings, both in the workplace and in the home.

While several indicators have been suggested for the detection of task-related changes in attention levels such as blink duration and rate [8], heart rate variability [9] and electroencephalographic measures [6] there is, however, no accepted “gold standard” technology for detecting attention decrement [7], aside from RT measures. Although some authors suggest that EEG-measured changes in brain activity might represent the most promising indicator of attention decrement [5], other studies have proposed that hybrid systems - based on the multimodal fusion of a number of indicators - may allow for more robust performance [10], [11]. In this vein, optical neuroimaging, namely Near Infrared Spectroscopy (NIRS), may represent a viable additional and complementary method for cognitive state monitoring. The purpose of this study is to address the issue by investigating the capability of this increasingly exploited neuroimaging method (i.e., NIRS), to detect real-time changes in brain activity related to decrements in the level of attention during a sustained attention task. In particular, this study investigates the sensitivity of (i) different NIRS-measured hemodynamic variables as well as (ii) different attention-related cortical areas to the attention decrement phenomenon.

NIRS is a versatile neuroimaging tool increasingly adopted in the neuroimaging-community [12], [13]. Ayaz *et al.* [14] assert that “*NIRS is safe, highly portable, user-friendly and relatively inexpensive, with rapid application times and near-zero run-time costs*” [15]–[19]. The modality has potential, as a portable measurement system for cognitive state monitoring outside the laboratory environment [20], [21]. Functional NIRS utilizes, as fMRI does, the tight coupling between neuronal activity and regional cerebral blood flow [22] to infer brain activation state from changes in oxy- (O_2_Hb) and deoxy-hemoglobin (HHb) concentrations characterizing the cortical hemodynamic response. Recently NIRS-derived cortical hemodynamic responses have been demonstrated to be sensitive to attention decrement during sustained attention tasks [23]–[30]. Further, it has been demonstrated through machine learning studies based on NIRS-measured hemodynamic variables (i.e., O_2_Hb and HHb) that the NIRS modality has some utility as a technology for active brain computer interfaces (e.g., [31]–[36]). Taken together, these findings suggest the potential of the technique as the measurement basis for an automated cognitive tracking technology. However, to date there have been no studies conducted to evaluate whether or not NIRS signals could be used for robust classification of different levels of attention during tasks requiring sustained attention. The primary object of past NIRS studies on sustained attention focused on better understanding the relationship between NIRS-measured cortical activity and degradation in behavioral performance. The current study aspires to go a step further by investigating the performance of a NIRS-based classification analysis aiming at distinguishing changes in the level of attention.

It is also worth noting that most of the aforementioned NIRS studies focused on one area of interest, the prefrontal cortex (PFC, [23]–[25], [27]–[30]). The PFC represents an appropriate candidate to investigate attention-related changes in brain activity since it has been described on numerous occasions as a cortical area significantly involved in human cognition (e.g., [37]). There are also convenient, practical benefits to mounting NIRS probes on this scalp area. One such benefit is that compared to other, more dorsal areas, the scalp in this region is hairless. Hair presents a well-known problem in NIRS as it can impact dramatically on both photon absorption and the coupling of the probes with the underlying scalp [38]. The associated optical losses can severely degrade the signal-to-noise ratio reducing the reliable interpretability of the signal. Another important benefit of PFC-oriented measurement is that by focusing on a single specific cortical area, the measurement setup is consistent with the aim of developing practical, ambulatory cognitive-state tracking technologies since it allows for a reduction in the number of measurement channels required at the scalp level. However, by investigating PFC activity only, NIRS studies in the field may miss other relevant information conveyed, potentially, by activity in other task-relevant cortical areas. Excluding information from such areas could limit the potential classification accuracy of NIRS-based classification of cognitive states. It is then crucial to investigate the potential of other attention-related areas’ activity, as measured through NIRS, to better capture the attention decrement. Lesion studies in patients [39] as well as neuroimaging studies in healthy subjects [40]–[42] suggest a significant role for the right parietal area in sustained attention processes and changes in activity under this area has been suggested as involved in attention changes [26], [43], [44]. This cortical region represents then another potential candidate for the discrimination of changes in the level of attention. Testing this hypothesis is an important aspect of the research described here.

Selecting the most discriminative variable(s)/feature(s) is an important aspect of any machine-learning problem [45]. Given that none of the aforementioned sustained attention studies performed any NIRS-based classification analysis, there are currently no guidelines concerning which NIRS variables to focus on in order to detect as accurately as possible any decrement in the level of attention. While some previous studies demonstrated that the HHb variable was insensitive to time-on-task during a sustained attention task (e.g., [28]), others have described significant changes in both the HHb and O_2_Hb variables throughout sustained attention tasks [23], [24], [46]. In the study described here, we hope to shed light on these discrepancies by investigating classification performance based on the O_2_Hb variable, the HHb variable and a combination of both.

In summary, as part of efforts to develop effective cognitive-state tracking technologies, this paper reports on a study investigating the potential of detecting attention decrements during a sustained attention task through optical measurement of related brain activity. Beyond this primary investigation, we also seek to test the hypotheses that (i) in addition to the PFC, other attention-related areas (i.e., the right parietal area) may facilitate the detection of attention decrement with good accuracy and (ii) exploiting both NIRS variable(s) (i.e., O_2_Hb and HHb) is valuable in improving performance for such efforts.

## Materials and Methods

### Participants

Seven male volunteers took part in this classification study (aged 29.0±6.6 years). All subjects were right-handed according to the Edinburgh Questionnaire [47]. None of the subjects reported that they suffered from neurological, respiratory, and cardiovascular disease or medication, which might affect brain perfusion and function. All procedures were approved by the local Institutional Review Board for the Protection of Human Subjects (CPP Sud-Méditerranée II, number 2010-11-05) and complied with the Declaration of Helsinki for human experimentations. Each subject provided written informed consent prior to participation.

### Experimental Set-up

Experiments were conducted in a quiet and dimly lit room. Each subject performed the entire protocol once. The subjects were seated at a table on which a stimulus light (white) source was positioned level with the eyes at a distance of 1 m. The left forearm of each subject was rested upon the surface of the table. The dominant hand (*i.e.*, right hand) was held in a neutral position in the sagittal plane. The angle of the elbow was set to 110° (with 180° corresponding to full elbow extension). The thumb was fixed against a dynamometer allowing direct measurement of abduction force (Captels, Saint-Mathieu-de-Tréviers, France).

### Experimental Protocol

**Figure 1:**
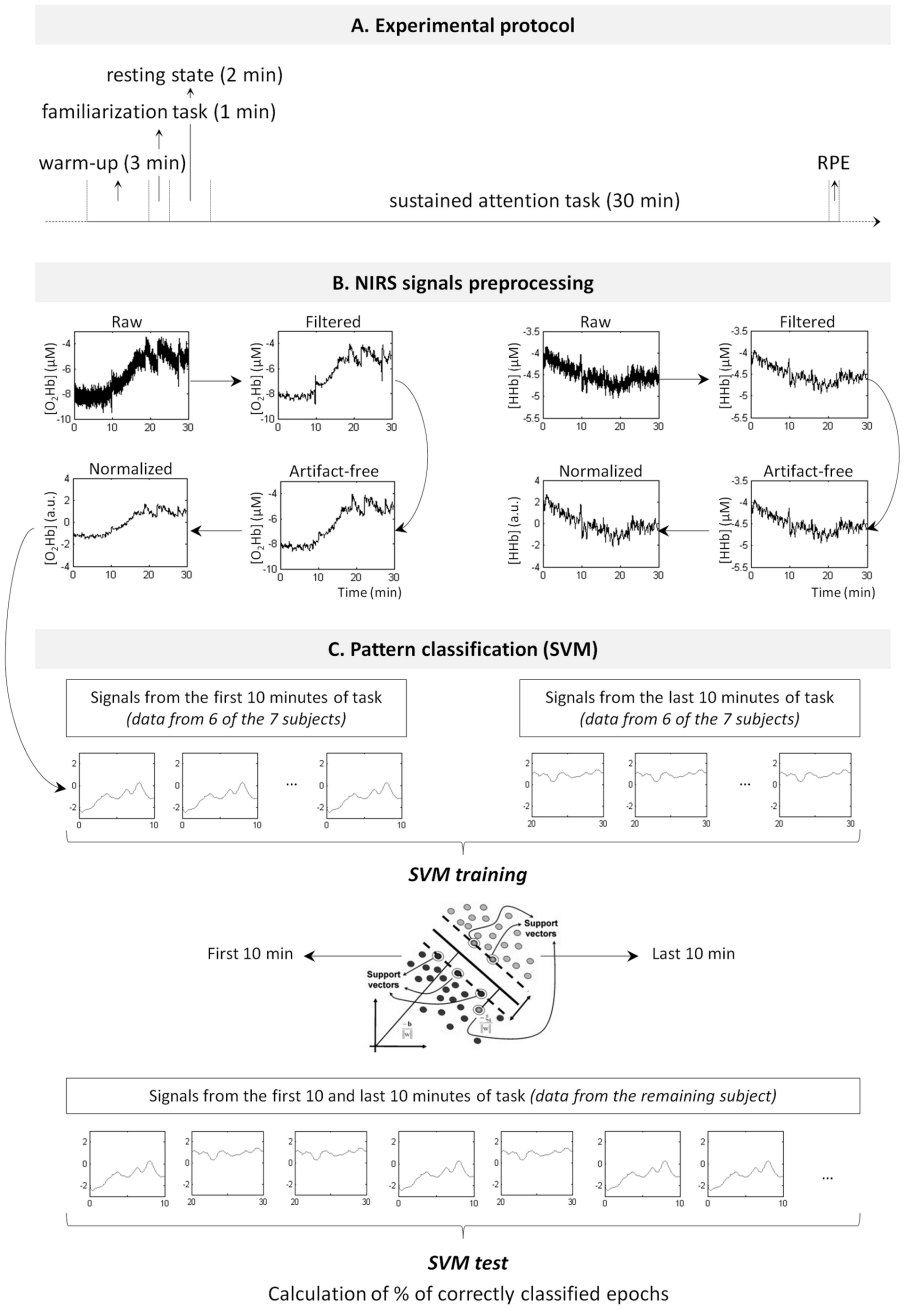
Illustration of the experimental protocol and analysis procedure. **A. Time course of the experimental protocol.** RPE: Rate of Perceived Exertion. **B. NIRS signals preprocessing steps.** *Left*: O_2_Hb signals. *Right*: HHb signals. **C.** **NIRS signals classification based on SVM.** *From top to bottom*: Filtered, artifact-free, normalized signals are first exploited in the SVM learning step using six of the seven subjects. A model is built, represented here by its feature space. Finally, a SVM test is performed using the signals of the single remaining subject and the percentage of correctly classified epochs is computed. See Methods for further details.

First, a standard warm-up phase was performed consisting of twenty static submaximal contractions of the right abductor pollicis brevis (*i.e.*, through a thumb abduction task) in an intermittent mode. The level of force was maintained for five seconds followed by five seconds of recovery and was gradually increased after the tenth contraction. Visual feedback of the level of force generated was given in real-time on a computer screen positioned in front of the subjects. Once the warm-up phase was realized, the computer screen was turned off and a simple visual RT task was performed over the course of one minute in order to familiarize the subjects with the paradigm. The task onset signal consisted of a flash (150 ms duration) delivered using the light source (i.e., photodiode arrays consisting of a few dozen emitters). A randomly varying inter-stimuli interval (ISI) was set with a range of between two and fifteen seconds. The motor response requested from the subject was a thumb abduction task to be performed as quickly as possible in response to the visual stimulus. In this sense, the task exploited in our protocol closely replicated the characteristics of the psychomotor vigilance test (PVT) developed by Dinges *et al.* [48]. Such a simple RT task has been shown to be highly sensitive to changes in attention [2], [48]. Further, during simple RT tasks, the stimulus saliency remains constant throughout the task and the maintenance of optimal performance is therefore only mediated through top-down processes without any stimulus-driven increase in the level of attention. Following the familiarization RT task, the subjects were instructed to rest for two minutes in order to produce a reference resting state in the NIRS signals. This was followed by a sustained attention task of thirty minutes whose characteristics were the same as those during the one minute familiarization task. Over the course of the experiment, event labels were set using the NIRS acquisition software (V6.0, Artinis, The Netherlands) in order to demarcate the periods of interest (*i.e.*, baseline and task). Immediately after the experiment, the rating of perceived exertion (RPE) was evaluated by means of the Borg scale (from 6 to 20; [49]). The time course of the experimental protocol is presented in Figure 1.A.

### Measurements

#### Reaction time

The force/motor responses and stimuli signals were synchronized and digitized at 2,048 samples per second using the Biopac MP100 data acquisition system (Biopac System, Inc., Santa Barbara, CA).

#### Near-Infrared spectroscopy

**Figure 2:**
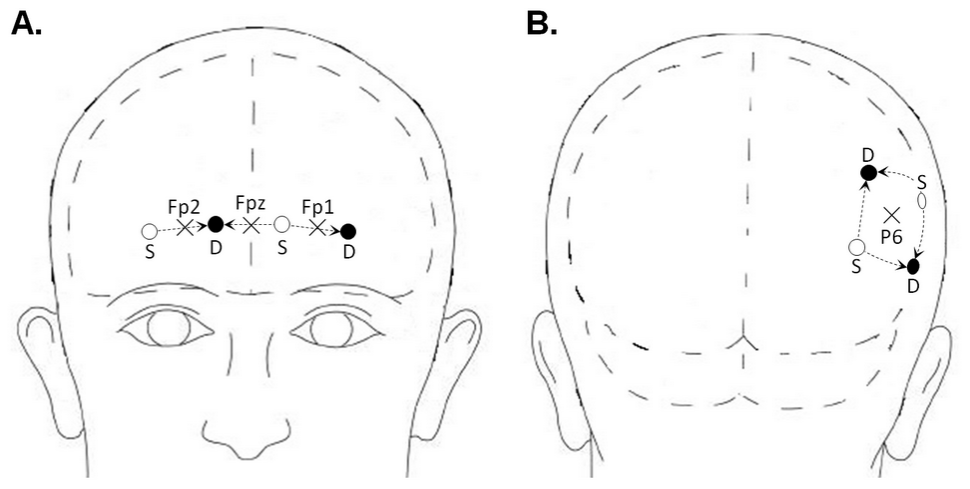
Placement of NIRS probes. Frontal (A) and dorsal (B) views are represented. Crosses represent locations from the EEG 10-10 system. Empty circles - noted “*S*” - represent sources and black circles - noted “*D*” - represent detector probes.

The NIRS technique has been described elsewhere [13]. NIRS measurements were performed using a continuous wave (CW) multichannel NIRS system (Oxymon Mark III, Artinis, The Netherlands). The data acquisition sampling rate was set to 10 Hz. This system allows measurement of changes in optical density at two different wavelengths in the near-infrared range (nominal wavelengths 763 and 855 nm) before converting these into changes in concentration levels of [O_2_Hb] and [HHb]. A subject-specific differential pathlength factor (DPF) was used for this conversion based on the age of each subject [50] and this allowed measurement of the concentration changes of [O_2_Hb] and [HHb] in µM [51]. The emitter-detector distance was set to 3.5 cm. In the present study, the measurements were performed using seven channels over the regions of interest. Three were positioned over the frontopolar part of the left, the right and the medial prefrontal cortex (lPFC, rPFC and mPFC, respectively), and four over the right parietal area. The probes were placed according to the modified international EEG 10–10 system [52] and mounted on a custom-made cap fixated by several bands surrounding the head of the subject. According to the EEG 10-10 system, the locations of the centers of the channels over the lPFC, rPFC and mPFC corresponded to the Fp1, Fp2 and Fpz points, respectively. The centers of the 4 channels set in a square template over the right parietal area corresponded to the P6 point. A representation of the channel locations can be seen in Figure 2. During the probe placement, the Oxysoft software (V6.0, Artinis, The Netherlands) allowed real time assessment of the quality of the NIRS signals for each of the seven channels based on the light source power level and the receiver gain. Once an acceptable signal-to-noise ratio was obtained according to the signal quality assessment, a zero baseline was set and the protocol was executed.

### Data Analyses

#### Behavioral data

The RT data was processed through the Acknowledge software associated with the Biopac system (Acknowledge 3.8.1, Biopac Systems, Santa Barbara, CA, USA). The RT was measured as the time between the flash stimulus (target stimulus) and the beginning of force production. Responses were considered correct if the dynamometer was engaged between 150 and 600 ms after stimulus onset. All other responses were considered incorrect. Such a cut-off time window has been exploited in other RT studies (e.g., [53]) and facilitates the exclusion of outlying RT values in the dataset. We calculated RTs of the first ten and last ten minutes of the task and then computed averages of the RTs obtained for these two periods.

#### Near-Infrared spectroscopy

Signal preprocessing: The oxy-and deoxy-hemoglobin signals acquired from the NIRS instrumentation were initially filtered using a fourth order digital low-pass Butterworth filter with a cut-off frequency of 0.1 Hz in order to remove the heart rate and respiratory components [54]. Next, movement artifacts were removed on specific, visually identified channels by using moving standard deviation and spline interpolation routines in Matlab (Mathworks, Natick, MA). This method has been validated using simulated, as well as real NIRS signals and has been shown to improve the detection of evoked hemodynamic responses (see [55], for details). Finally, given that the datasets contained information regarding cortical hemodynamic changes over several regions of the brains and from many different subjects, a z-normalization of the signals was performed. From the resulting signals, a supervised classification procedure was performed by means of a linear support vector machine (SVM) algorithm.

NIRS data classification using support vector machines: SVM can be considered as one of the most powerful classification algorithms as it is able to learn linear decision boundaries as well as more complex ones with relatively low complexity and few user-defined hyper parameters [56]. Nonlinear decision boundaries are learned using the “kernel-trick” which consists of mapping the data into a higher-dimensional space using a kernel function and finding a linear separation in that space. An example of kernel function is the Radial Basis Function (RBF) defined as follows:
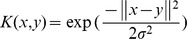



Where *x* and *y* are two data points and 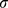 is the width of the RBF.

**Figure 3:**
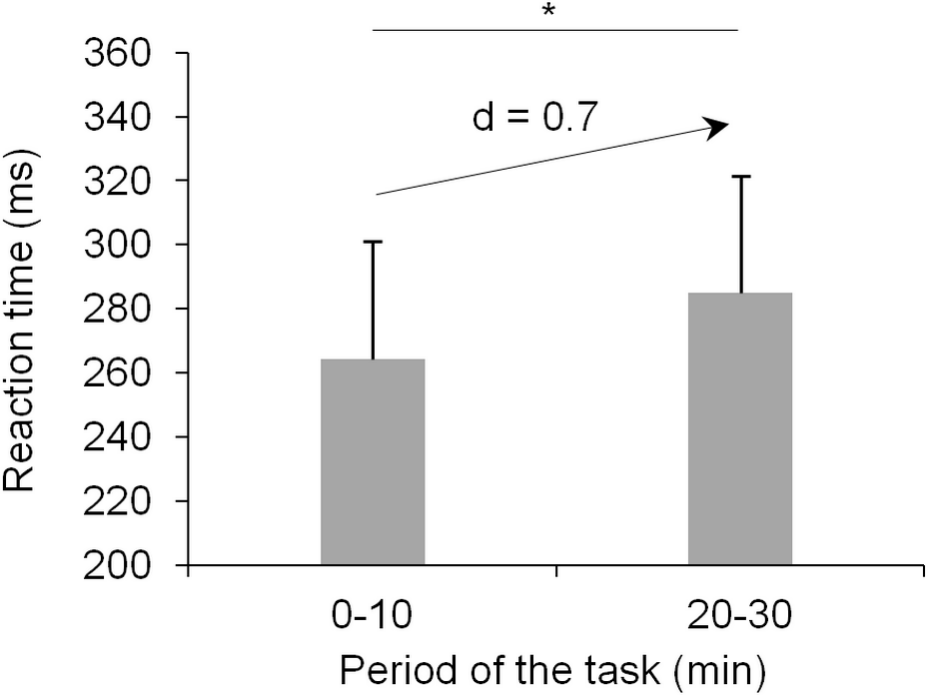
Changes in mean reaction time from the first ten to the last ten minutes of the task. A significant increase in RT occurred at the end (last ten minutes) compared to the beginning (first ten minutes) of the task. Cohen’s effect size d value for this difference is specified above the centered arrow. *p<.05. Vertical bars represent SD.

In the current study, we used a linear SVM (i.e., using linear kernel) as the decision boundary between the two brain states (i.e., full attention vs. attention decrement) appeared to be sufficiently linear. The linear SVM has already been used in previous studies on attention decrement detection based on EEG signals, illustrating high classification accuracy results (e.g., [7]). Like other linear classifiers (e.g., linear discriminant analysis, LDA), a linear SVM uses a hyperplane to separate data points from each class. Additionally, the linear SVM chooses the hyperplane with the maximal distance from the nearest training points. This distance is called the “margin” and the nearest training points to the optimal hyperplane are called “support vectors”. Figure 1.C. shows an illustrative example of an optimal hyper-plane as constructed by a linear SVM. Margin maximization increases generalization ability of the classification algorithm. However, such a learning scheme is sensitive to outliers and overtraining. For this reason, a regularization parameter *C* is used to reduce data over-fitting. Depending on *C*, the optimal margin will either expand or diminish and more or less points will subsequently become support vectors, respectively [56], [57]. In the current study, we used the default value of 1 for the regularization parameter *C* with the software Weka (version 3.6.8, University of Waikato Hamilton, New Zealand). We designed the SVM for two-class classification (i.e., full attention versus attention decrement). The NIRS signals from the first ten minutes of the task were considered as characterizing the ‘*full attention*’ class, while the NIRS signals from the last ten minutes of the task were considered as characterizing the ‘*attention decrement*’ class (this assumption was then supported by analyzing the RT values as described below; see RT results, Figure 3). Classification analyses were performed over data segmented and averaged over one second duration epochs. Thus, for each subject, six hundred time points were obtained for each class (i.e., sixty seconds×ten minutes) and constituted the corresponding point clouds within the feature space. The signal feature selected was the magnitude (i.e., averaged for each one second duration epoch) of concentration values (in µM) of the considered NIRS variable(s): [O_2_Hb], [HHb] or both [O_2_Hb] and [HHb]. Also, classification was based on the NIRS signals from (i) the PFC area exclusively, (ii) the right parietal area exclusively and (iii) both the PFC and the right parietal areas. These distinct feature vectors allowed us to investigate classification accuracy over a range of NIRS variables and cortical area(s). By doing so, the resultant feature pool was comprised of between three (i.e., using [O_2_Hb] or [HHb] from the three channels over the PFC) and fourteen features (i.e., using both [O_2_Hb] and [HHb] from the seven recorded channels). Data obtained from six of the seven subjects were exploited as the training set. Once the training step was realized, each class of the resulting feature space consisted of three thousand six hundred points (i.e., six hundred points×six subjects). The test data set consisted of the data of the remaining subject. This process of leave-one-out cross-validation was repeated to assess the classification accuracy across all subjects. Classification accuracy was calculated as the percentage of correctly classified epochs for each part of the data (i.e., first or last minutes of task). All of the processing steps are presented in Figure 1.

### Statistical Analysis

Statistica software (version 7.0, Statsoft, Oklahoma, United-States) was used for all analyses. All data were examined for normality using skewness and kurtosis tests. The Student t-test was used to test for any significant effect of time (i.e., first ten versus last ten minutes of the task) on the changes in RT. Effect size was calculated on the RT values using Cohen’s effect size d (d effects: small ≥0.2, medium ≥0.5, large ≥0.8), defined as the mean change score divided by the standard deviation of change [57]. The significance level was set at p<.05. Data are presented as mean ± standard deviation (SD).

## Results

### Behavioral Results

As expected, the RT results demonstrated that attention decrement occurred towards the end of the task. The Student t-test demonstrated that RT values were significantly higher in the last ten than for the first ten minutes of the task (t_6_ = 3.1; p<.05). The Cohen’s effect size d value for this difference was 0.7, corresponding to a medium-to-large effect. These results are presented in Figure 3. The RPE score after the experiment was 14.9±1.7, a value corresponding to “hard” according to the scale.

### Classification Accuracy

**Figure 4:**
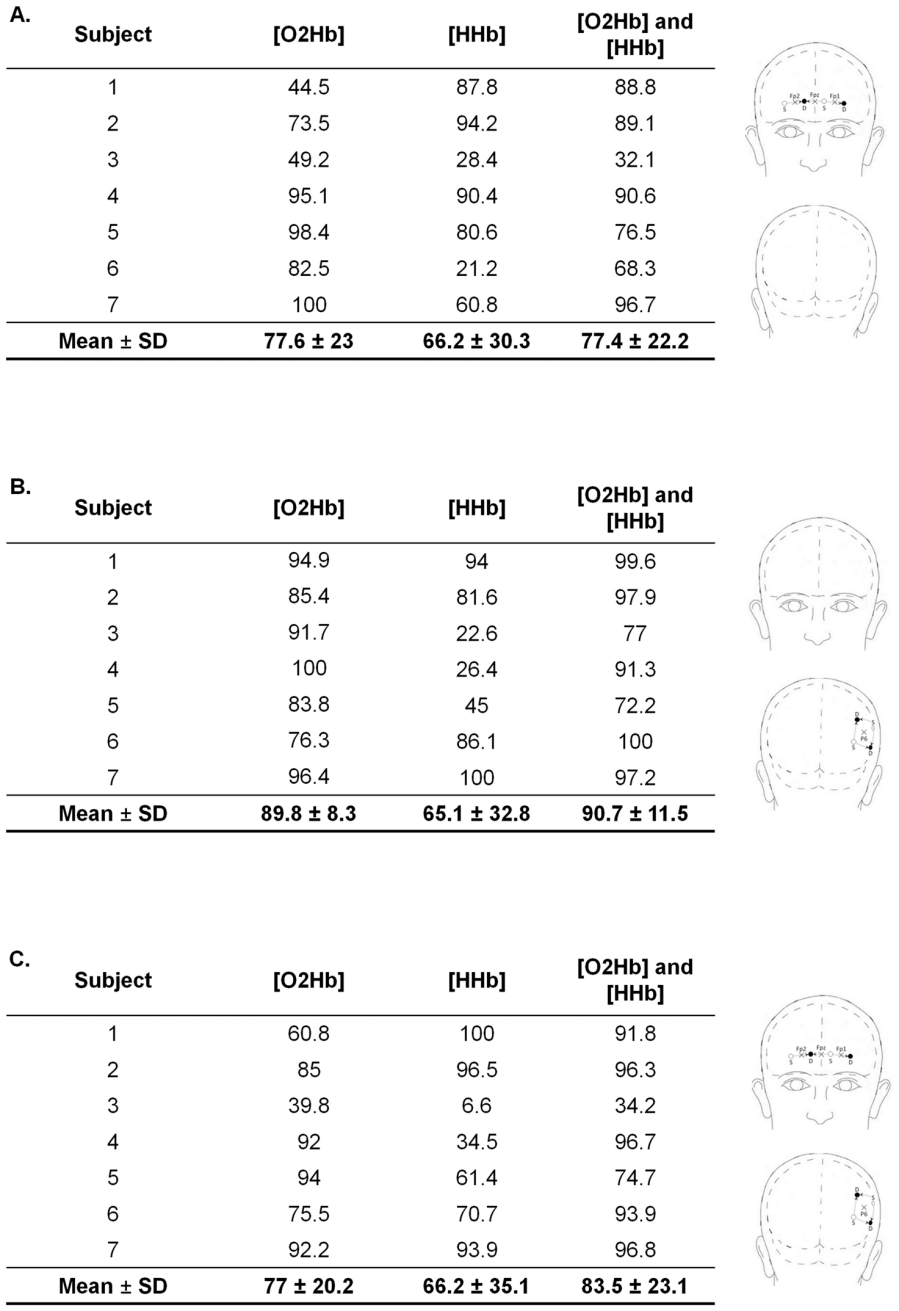
Detailed classification accuracy results. The classification accuracy results using NIRS signals from the prefrontal (A), the right parietal (B) and both the prefrontal and the right parietal areas (C) are provided. From left to right, the columns present (i) subject number, the classification accuracy - in percentage of total classification trials - exploiting (ii) [O_2_Hb], (iii) [HHb] and (iv) both [O_2_Hb] and [HHb] as features of interest.

All the classification accuracy results, including analyses exploiting [O_2_Hb], [HHb] and both [O_2_Hb] and [HHb] as features of interest from the PFC area exclusively, the right parietal area exclusively and both the PFC and the right parietal areas, are presented in Figure 4. The main results indicate that (i) the highest classification accuracy results were obtained when exploiting the oxyhemoglobin signals (i.e., from 77 to 89%, depending on the cortical area considered) rather than the deoxyhemoglobin signals (i.e., from 65 to 66%) and (ii) the classification accuracy was increased to about 90% when using signals from the right parietal area rather than from the prefrontal cortex.

## Discussion

This study aimed to investigate the potential of harnessing NIRS-measured cortical activity for the detection of time-on-task related changes in the level of attention during a sustained attention task. Our experimental design produced a decrement in the level of attention as revealed by the significant increase in RT in the last ten compared to the first ten minutes of the task (p<.05; d = 0.7). The results demonstrate that relatively good classification accuracy can be obtained using NIRS variables (O_2_Hb and/or HHb) to detect the changes in the attentional state observed at the behavioral level. It is worth noting that the classification accuracy was lowest when exploiting the HHb signals only (i.e., from 65 to 67% in average), regardless of the cortical area considered. Moreover, the classification accuracy was increased to about 90% when using signals from the right parietal area. These findings are examined in detail next.

### Methodological Considerations and Study Limitations

As already mentioned, the task studied here closely replicated the characteristics of the psychomotor vigilance test (PVT) developed by Dinges *et al.* [48] and an increase in RT has been previously demonstrated for such a PVT, even for a task of twenty minutes duration [2]. As expected, a significant increase in RT occurred during this simple RT task of thirty minutes duration. In addition to the longer RT, relatively high RPE values were reported by the subjects (i.e., 14.9±1.7 on a scale ranging from 6 to 20), which demonstrates the cognitive loading (sustaining of attention) demanded with such a simple sustained attention task [58]. Also, Lim *et al.* [2] have shown that the increase in RT observed during their simple RT task of twenty minutes duration was accompanied by high subjective fatigue ratings after the task - subjective fatigue being well-known to affect sustained attention abilities [59]. Taken together, these results support the conclusion that the task exploited in the current study induced a time-on-task related attention decrement.

As discussed by Shen *et al.* [7], one weakness of past studies exploiting EEG to detect attention decrement was the lack of subject-wise cross-validation in their performance evaluation (e.g., [60]). We therefore applied, as in [7], a “*leave-one-out*” scheme (which is a conventional approach to evaluate the performance of machine learning methods for small data sets) in order to evaluate the subject-independent accuracy performance. Using this leave-one-out cross-validation procedure, high classification accuracy was confirmed with up to 90% scores achieved in classifying attention state based on NIRS signals. The use of such a cross-validation procedure was particularly appropriate in this study as we had a relatively small number of subjects (n = 7), a small sample size which may be considered a study limitation. It is worth adding the caveat that leave-one-out schemes may induce, for small sample sizes, a bias in the error estimation [61]. Using a larger number of subjects would facilitate the exploitation of other validation schemes such as *k*-fold cross-validation which may afford less biased estimations of the model generalization error.

Another potential issue in the current study was the lack of control for any skin flow contributions to the NIRS signals and again, this may be regarded as a study limitation. Recent studies have raised the issue of superficial - extra-cortical - contributions in NIRS signals, specifically in the O_2_Hb signal [62]. The analysis of the photon time-of-flight distribution in time-domain NIRS [63], [64] or the use of additional short emitter-detector separation as regressors [65], [66] have been proposed as methods to separate cortical and extracortical contributions in NIRS signals. In the study described here, the clear variability in attention decrement-sensitivity across the cortical areas investigated does not support the idea of a global systemic response biasing the feature space. The observed increased activity from areas known to be involved in attention suggest that the features identified reflect localized cortical vascular dynamics. The use of the aforementioned methods would have however helped identify the precise nature of the contribution from cortical components in the optical signals obtained.

The regional variation in the differential path-length factor (DPF) identified in the literature [67] might also have affected the measured regional changes in NIRS signals. We exploited a subject-specific DPF based on the age of each subject as proposed by Duncan et al. [50] and this allowed the measurement to be converted into changes in concentration levels of [O_2_Hb] and [HHb]. In order to eliminate the heterogeneous effect of regional DPF variations across the full extent of the measurement area, the signals were normalized through expression in terms of percentage changes (Figure 1.B.). Future NIRS investigation might however implement region-specific DPF in addition to subject-specific DPF.

Finally, our classification procedure specifically aimed at classifying attention decrement-related lapses in attention, as they occur during time-on-task activities. To do so, we exploited the first and last ten minutes of the task to label our classes. An alternative means to detect changes in the level of attention could involve labeling the brain states of interest using moment-to-moment variations in behavioral performance (*e.g.*, RT). Doing so would facilitate the detection of changes in the level of attention on shorter time scales, as they occur momentarily, but such an approach is beyond the scope of the current study.

### Region of Interest: Right Parietal Area Versus PFC

This study aimed, in part, at testing the appropriateness of focusing on the PFC to detect decrements in the level of attention. The emphasis on the PFC which has characterized research to date in this field [23]–[25], [27]–[30] has probably been as a consequence of an *a priori* knowledge-driven choice (i.e., the PFC area has been identified as involved in a large number of cognitive functioning studies) and because of technical advantages that presents this hairless scalp area conveniently for NIRS investigation. In contrast, our experimental investigation has taken a data-driven approach to deduce which of the attention-related cortical areas offers the best classification accuracy when investigated using NIRS. We hypothesized that, given its implication in sustained attention tasks, the right parietal area would represent another potentially relevant candidate area over which to discriminate changes in the level of attention.

For both analyses, based on the PFC or on the right parietal area signals, relatively good classification results were obtained, however performance was on average much better when exploiting NIRS signals recorded over the right parietal area (see Figure 4.B). This finding is not surprising when one considers the aforementioned, crucial role of this area in sustained attention tasks [39]–[42]. This result raises design dilemmas for NIRS-based cognitive-state tracking technology: would it be preferable to focus on the right parietal area – which yields better discrimination and reduces then the possibility of false positives in the detection of attention decrement? Or rather, would it be better to continue to focus on the PFC which offers undeniable technical advantages for NIRS investigation, but offers poorer sensitivity to attention decrement? Although the presence of hair over the parietal area did not impact the classification accuracy results of our study, it was technically more complex, and hence took more time to set up than when measuring over the PFC. The problem of obtaining qualitatively good NIRS signals over hair-covered scalp areas is well-known from NIRS investigators and has been identified as an issue in motor area-based brain-computer interface design [16]. The challenge for future NIRS technological developments is to provide a NIRS optode mounting system which can resolve this problem of hair-related photon absorption. In such conditions, focusing on the right parietal area for detecting attention decrement in a real world context could become more convenient. It is worth commenting too that the positioning of probes over the parietal cortices is likely to be much more acceptable to users given that it is aesthetically less intrusive than the alternative which would require the mounting of a set of optodes and sensing technology on the face (i.e., the forehead).

Finally, it is worth commenting on the finding that combining signals from both the PFC and the right parietal areas did not improve classification performance accuracy over the use of features from the right parietal area only in our classification analyses. This result potentially indicates that, rather than being additive or even multiplicative, information extracted from neural signatures of attention decrement over the PFC and right parietal areas may be redundant. In the purpose of developing practical, ambulatory cognitive-state tracking technologies, we previously mentioned that the number of measurement channels required at the scalp level should be minimized. Thus, the right parietal area may, on its own, represent an appropriate measurement area for NIRS-based detection of attention decrement.

### Variable of Interest: O_2_Hb versus HHb

The second objective of this study was to determine which NIRS variable(s) should be exploited for the purpose of distinguishing changes in the level of attention. As mentioned in the introduction, some authors failed to find any changes in HHb in response to time-on-task, even during a sustained attention task of 3 hours duration [28]. The classification results here support the results of Li *et al.* [28], and provide further evidence that the HHb variable has poor sensitivity to time-on-task related changes in the level of attention. This result can be explained by the existence of smaller changes in HHb compared to that in O_2_Hb during neurovascular coupling - a phenomenon well “represented” by the balloon model [68]. Also, changes in O_2_Hb have been described to more directly reflect cortical activation than HHb due to its superior contrast-to-noise ratio [69], and previous NIRS studies have even proposed that researchers should focus on O_2_Hb - rather than HHb - as the variable of interest to determine changes in cortical activity [26], [70].

The combination of both O_2_Hb and HHb variables in our classification analysis, in some cases improves performance. This increase is minor when measured over the right parietal area (about 1%) and the combination of variables in that example is of minimal utility. As one problem in developing useful cognitive-state tracking technologies is that of reducing its computational requirements [6] here again, a choice has to be made between two alternatives, that is: either (i) exploiting both the O_2_Hb and the HHb variables as features of interest in order to marginally improve performance at the cost of increased computational overhead (i.e., by doubling the dimension of the feature space) or (ii) focusing on the O_2_Hb to reduce the computational cost with a minor loss in classification performance. In our opinion, the latter alternative appears to be the more appropriate choice for the purpose of future real-time applications although it depends on the precise use-case envisaged.

### Conclusion and Perspectives

To the best of our knowledge, the present study is the first to describe an approach to detect changes in the level of attention through monitoring hemodynamic signals and the results may serve as a further step towards the development of a NIRS-based cognitive state tracking system. Our data-driven approach leads to the conclusion that (i) the right parietal area represents a better choice for the positioning of optodes as it is less intrusive and more sensitive than the PFC and (ii) the O_2_Hb variable appears to be sufficiently sensitive for characterization of attention decrements as they occur in cortical areas. The results also demonstrate that optical neuroimaging constitutes a relevant method of significant potential for cognitive state monitoring. We feel the method may have most benefit through integration within a hybrid system context where a combination of complementary modalities (e.g., EEG and NIRS) may provide more robust performance over each modality used in isolation.
